# High performance hierarchical porous carbon derived from distinctive plant tissue for supercapacitor

**DOI:** 10.1038/s41598-019-53869-w

**Published:** 2019-11-21

**Authors:** Jinxiao Li, Yang Gao, Kuihua Han, Jianhui Qi, Ming Li, Zhaocai Teng

**Affiliations:** School of Energy and Power Engineering, Shandong University, 250061 Jinan, P.R. China

**Keywords:** Supercapacitors, Porous materials, Structural properties

## Abstract

It is generally acknowledged that the activation method and component of the precursor are of great importance for making porous carbon. In this study, four plant materials belong to one genus were selected as optimized plant material to produce hierarchical porous carbon for supercapacitors, the influence of initial structure was discussed. All the produced porous carbons have large specific surface area (higher than 2342 m^2^ g^−1^), high microporosity (more than 57%), and high pore volume (larger than 1.32 cm^3^ g^−1^). All the samples show characteristic of electrical double layer capacitance, and the onion-based porous carbon obtain highest specific capacitance of 568 F g^−1^ at the current density of 0.1 A g^−1^. With the current density rising from 1 A g^−1^ to 50 A g^−1^, the specific capacitance only decreases for 20%. After 5000 cycles, all the samples show relatively high capacitance retention (up to 97%). Two-step acid pickling has washed most impurities and directly lead to small equivalent series resistance (lower than 0.2 Ω). The samples show high power density and energy density (71 W h kg^−1^@180 W kg^−1^, 210 kW kg^−1^@33 W h kg^−1^). This study open an avenue to create high-performance hierarchical porous carbon based on plant architecture.

## Introduction

Supercapacitor, as one of the most promising energy storage device, has many peerless advantages than batteries, such as high power density, good cycle performance and high rate performance^1–3^. Electrode material is the key component of supercapacitor, and porous carbon (PC) is one of the most popular electrode material^4,5^. But the high cost of the raw material is a constraint for batch production^6^. Biomass, as one kind of renewable carbon source, has wide distribution, large quantity and cheap price, which attracted tremendous attention in recent years^7–11^.

Generally, the precursor for PC is supposed to have higher carbon content and lower ash content, which will lead to higher yield and higher quality. In addition, some elements also play important parts during the preparation process. N, O and P can affect the electrochemical performance by forming corresponding surface functional groups^12,13^. The embedded alkaline or alkaline earth elements^4,14,15^ in the biomass will facilitate the pore-forming process during carbonization^16–18^.

It is generally accepted that PC with hierarchical pore structure has higher capacitance and better rate capability than those with homogeneous pores^19^. It can be attributed to the different functionalities of different pores: macropores (>50 nm) can store electrolyte, mesopores (2–50 nm) can shorten the transport distance of the ions, and micropores (<2 nm) are the very part to store electric charge^20^. Therefore, the amount of micropores can affect electric capacity to a certain extent.

Many studies have already proved that some special structures of biomass precursor can be kept after preparation process. Cheng *et al*.^21^ found that the carbon aerogel made from degreasing cotton still kept the original appearance of cotton fiber. Zhang *et al*.^22^ found that after carbonization and activation, some initial microstructures of garlic skin can still be reserved. Liu *et al*.^23^ found that some morphology of perilla leaf can be preserved after carbonization. Higher plants have mature tissue differentiation, and they have natural micro-sized hierarchical transportation structure for water, nutriment, and ions. Therefore, a more developed hierarchical structure can be constructed by introducing a certain amount of activate agent based on its natural structure.

For a long time, it was generally believed that shells are the best biomass precursor for PC^24–26^. However, some studies recently found that PC derived from other plant organs with specific structure show better performance^8,16,23,27,28^. Although the precursor mentioned above are from different organs, they share some common tissues. There should be some specific tissue with both good structure and component advantages for PC. However, few studies unveil the connection between the PC structures and plant tissues.

**Figure 1:**
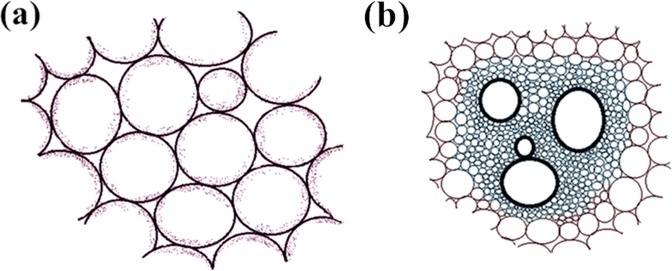
The microstructure of two special plant tissue. (**a**)parenchyma (**b**) vascular bundle.

Parenchyma (Fig. 1a) is one of the most common tissue with thin cell walls and the cells are loosely distributed^29^. It is different with sclerenchyma tissue and collenchyma tissue, which are abundant in fruit shells and made up by lignified sclerenchyma cells. Compared to the other two tissues, the thin cell walls of parenchyma means it has low mechanical strength and can be easily destroyed. As can be seen from Fig. 1a, obvious initial pores can be observed because the loose arrangement of the cells. The specific structure makes it easier for activator to take effect deep into the internal of the precursor, and the hierarchical pore structures can be created. Vascular bundle (Fig. 1b) is also a special tissue because it is natural channel for nutrient transportation^30^. Therefore, it can be used as transportation channels for electric charge.

The purpose of this study is to verify whether parenchyma and vascular bundle-riched plant material are superior for preparing hierarchical PC. KOH activation was applied and a two-step acid pickling was used for removing impurities in carbonization and activation process.

## Experimental

### Selection of raw materials

**Figure 2:**
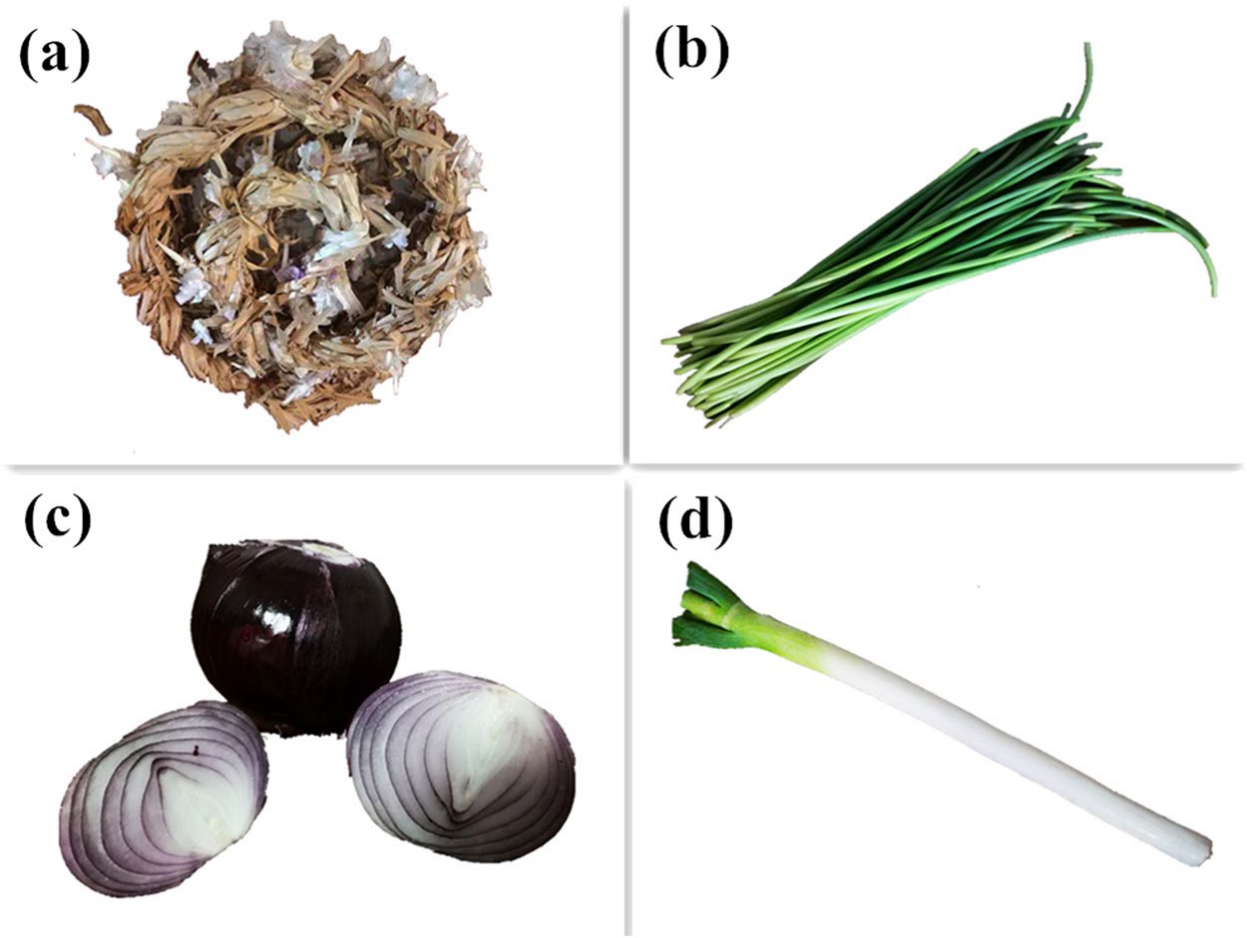
4 different plant material and the morphology of its microstructure. (**a**) Garlic seedling (**b**) garlic sprout (**c**) onion (**d**) scallion stalk.

Four different plant material (Fig. 2) which are rich in parenchyma and vascular bundle, were selected as raw material. All the plant samples are belong to allium of liliaceae, and they share similar structures and components. The 4 plants are garlic seedling (the seedling of a garlic plant), garlic sprout (the inflorescence of a garlic plant), onion and scallion stalk, and the produced PC are written as GSPC, GPC, OPC and SPC.

### Preparation of porous carbons

The raw materials were washed and dried at 105 °C for 24 h, smashed and screened through 80 mesh sieve. The powder was put into a tubular furnace for carbonization at 600 °C for 2 h with the heating rate of 5 °C min^−1^.The carbide was washed by HCl and deionized water till pH = 7. After drying in an air dry oven, the carbide was mixed with KOH with a mass ratio of 1:4, and the mixture was then put into a muffle furnace for activation at 800 °C for 2 h. Nitrogen was used as shielding gas through the process of carbonization and activation, and the flow rate is 0.5 L min^−1^. Both of the carbonized and activated product was pickled by 1 M HCl and washed by deionized water until pH = 7.

### Characterization methods of porous carbons

The surface morphology of the materials were observed by scanning electron microscopy (SEM, Carl Zeiss AG., Supra 55). Pore structure was characterized by N_2_ adsorption-desorption isotherms at 77 K (JWGB SCI.&TECH., JW-BK132F). The SSA was calculated by Brunauer-Emmett-Teller (BET) method, the mesopore volume and pore diameter were calculated by Barrett-Joyner-Halenda (BJH) method, and the data of micropores were calculated by Horvath-Kawazoe (HK) method. X-ray diffraction (XRD) and Raman spectra were used for analyze the graphitization degree of the PC samples. Energy dispersive spectroscopy (EDS) and X-ray photoelectron spectroscopy (XPS) measurement were carried out to investigate element components in the samples.

### Electrochemical Characterization

Porous carbon, conductive graphite and polytetrafluoroethylene (PTFE) were mixed with the mass ratio of 8:1:1. Moderate absolute ethyl alcohol was used as solvent. The mixture was treated by ultrasonic dispersion for 30 min, and the slurry was loaded on circular nickel foam. After drying in a vacuum oven at 80 °C for 12 h, the electrode slice was pressed under 12 MPa for 60 s. A button cell was assembled for electrochemical test, and 6 M KOH was used as electrolyte. Electrochemical test was carried out on a CS310H electrochemical workstation.

Specific capacitance (C, F g^−1^), energy density (E, W h kg^−1^) and power density (P, W kg^−1^) were calculated by following equations:

1 $${\rm{I}}=\frac{i}{0.8\,m}$$

2 $${\rm{C}}=\frac{4I{\rm{\Delta }}t}{V}$$

3 $${\rm{E}}=\frac{C{V}^{2}}{8}$$

4 $${\rm{P}}=\frac{E}{{\rm{\Delta }}t}$$

where *I* (A g^−1^) is the test current density, *i* (A) is the test current, *m* (g) is the total mass of both the electrode, *∆t* (s) is the discharging time, *V* (V) is the potential difference before and after discharge.

## Results and Discussion

### Characterization of pore structures

**Figure 3:**
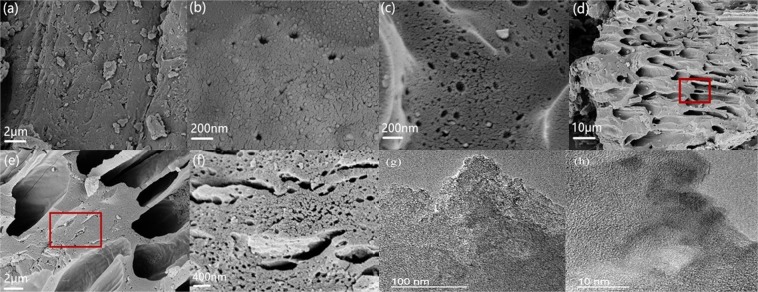
The morphology of the raw material, carbonized sample and activated sample. (**a**) Garlic sprout powder (**b**) carbonized garlic sprout without acid pickling (**c**) carbonized sample after acid pickling (**d**) activated sample after acid pickling (**e**) enlarged image of marked area in (**d**,**f**) enlarged image of marked area in (**e**,**g**) TEM image of the GPC (**h**) high resolution TEM image of the GPC.

As all of the four plant materials share many common properties in structure and component, to simply the presentation, garlic sprout was chosen as a typical sample to study the relations between the structure of PC and the initial plant tissue. As we can see in Fig. 3a, the raw material has initial pore structures on the smooth surface, and the shape of the pores are irregular. These structure is related to the initial pores in Fig. 1a. After carbonization (Fig. 3b), some tiny cracks start to appear and some circular pores are generated. After acid pickling, more pores are formed because some impurities are removed (Fig. 3c).It can be seen clearly from Figs. 3d,e that the structures of vascular bundle can be preserved after activation. That is because vascular bundle has physiological function to support plant, so it has enough strength to avoid the disruption during carbonization and activation. With the increase of the magnification (Fig. 3f), abundant pores with different shapes and sizes can be observed on the surface of the material. Abundant mesopores and micropores can be observed in the TEM images (Fig. 3g). Ordered lattice arrangement can be observed in Fig. 3h, which means the GPC has a certain graphite layers. A conclusion can be drawn from Fig. 3 that the structure of vascular bundle can be inherited after activation, and hierarchical porous structure, which has huge potential for charge storage, can be created based on the vascular structure. What’s more, the good structure of the precursor also has the potential to form ordered graphite structure.

**Figure 4:**
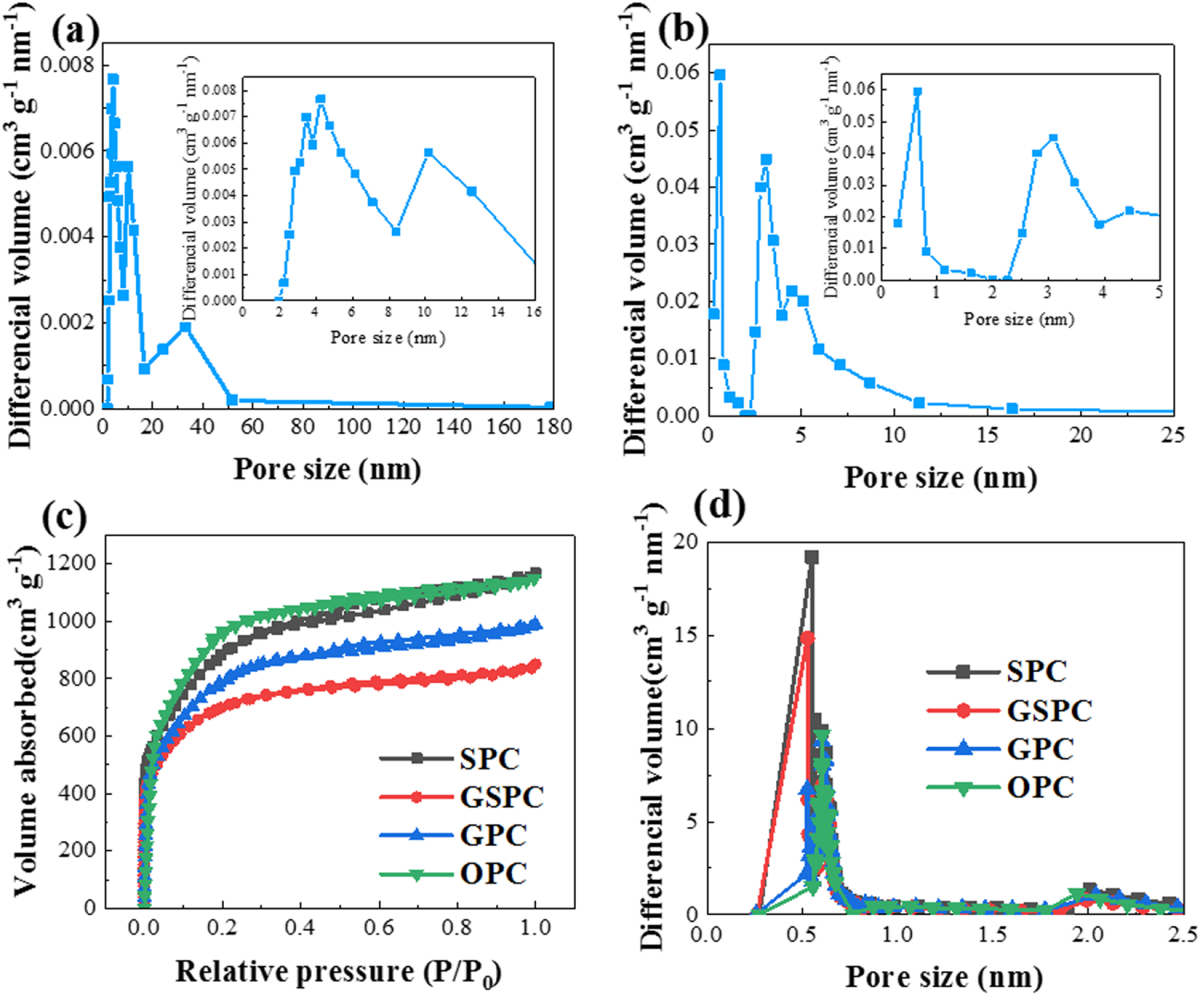
Pore characterization of 4 PC samples. (**a**) Pore size distribution of carbonized garlic sprout before acid pickling (**b**) pore size distribution of carbonized garlic sprout after acid pickling and washing (**c**) N_2_ adsorption-desorption isotherms at 77 K (**d**) pore size distribution obtained by BJH method (mesopore) and HK method (micropore).

The preparation progress can be further explained by Fig. 4a,b and Table S1. After carbonization, the SSA only reaches 7.70 m^2^ g^−1^. The pores are composed of mesopores and macropores according to Fig. 4a, and no micropores are detected according to the enlarged view. After acid pickling and washing, the SSA reaches to 56.26 m^2^ g^−1^, which is in accord with Fig. 3c, and pore volume increases from 0.084 cm^3^ g^−1^ to 0.116 cm^3^ g^−1^ (Table S1). It further proves that acid pickling and washing can remove impurities and create more pores at their position. Micropores can be detected at the moment, and the proportion of small mesopores are also improved. The pore structure of carbonized product is beneficial for the following activation, because activator can easily penetrate into the pores based on the larger contact area and more pores can be created.

Pore structures of four different PCs were further characterized by N_2_ adsorption-desorption experiments. Figure 4c exhibits N_2_ adsorption-desorption curves of 4 PC samples. According to the IUPAC classification, the four curves are all in accordance with type IV isotherm^31^. Hysteresis loops can be observed when the relative pressure (P/P_0_) is between 0.4 to 1.0, which proves the existence of different types of pores and the absence of blocking effect during desorption^32^. The curves are almost horizontal at high relative pressure region, and major adsorption occurs under P/P_0_ = 0.1, which means these PC samples possess high microporosity^33^. Combined with Fig. 4d, a narrow pore size distribution can be observed in the range of 0.5–0.7 nm, which confirms that all the PC samples are rich in micropores. It has already been proved that at a low charge-discharge rate, the pores with a size between 0.4–1.0 nm can still contribute to the capacitance through the desolvation of ions^34,35^. Some mesopores (pore size ~2 nm) also can be observed in Fig. 4d, these mesopores can store electrolyte, shorten the distance of electrolyte diffusion, and supply electrolyte in the process of charging and discharging^36^.

The details of pore structures for these four PC samples are shown in Table 1. Both the SSA and total pore volume of the PC samples has increased rapidly after activation and acid pickling compared with carbonized samples. All the PC samples have high SSA above 2300 m^2^ g^−1^, and large total pore volume above 1.32 cm^3^ g^−1^, which is better than most samples^8,24,37–41^. The micropores of the four samples occupies over 70% of total pore volume, which indicates their large potential for charge storage.

**Table 1:** Characteristics of pores in four PC samples.

Sample	SSA (m^2^ g^−1^)	V_T_ (cm^3^ g^−1^)	V_micro_ (cm^3^ g^−1^)	D (nm)	D_micro_ (nm)
GSPC	2370	1.32	1.02	2.22	0.64
GPC	2659	1.42	1.04	2.13	0.64
OPC	2342	1.55	0.88	2.65	0.64
SPC	3154	1.81	1.26	2.29	0.65

V_T_: total pore volume; V_micro_: micropore volume; D: average pore size; D_micro_: average micropore size.

### Electrochemical performance

**Figure 5:**
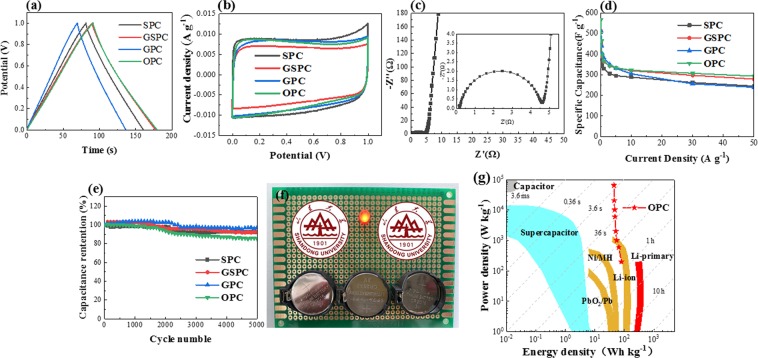
Electrochemical performance of PC samples. (**a**) GCD curves of four PC samples at current density of 1 A g^−1^ (**b**) CV curves of four PC samples at scan rate of 50 mV s^−1^ (**c**) Nyquist plot of SPC (**d**) specific capacitance of 4 PC samples at current density of 0.1–50 A g^−1^ (**e**) cycle performance of 4 PC samples by repeating GCD test at current density of 5 A g^−1^ (**f**) LED lighten by the supercapacitor device (**g**) Energy density and Power density of OPC.

The electrochemical performances of the four PC samples were measured by galvanostatic charge-discharge (GCD) test, cyclic voltammetry (CV) test and electrochemical impedance spectroscopy (EIS) test. The GCD curves of all the four samples are nearly isosceles triangle (Fig. 5a), which is typical characteristic of electrical double-layer capacitance (EDLC). The CV curves in Fig. 5b also present rectangle shapes with nearly no redox peak, indicating ideal EDLC behaviors.

As the PC samples share many similar properties, one PC sample will be chosen randomly in the following analysis. OPC was chosen for further GCD and CV test under different current density (0.1~100 A g^−1^) and different scan rate (5~200 mV s^−1^). As it can see from Fig. S1a, when the current density increases from 0.5 A g^−1^ to 10 A g^−1^, the curves still show good symmetry with nearly linear slopes within the voltage window of 0–1 V. The similar situation is happened in Fig. S1b. With the increasing of scan rate, the shape of CV curves are still in rectangular-like shape, indicating an obvious EDLC character with good rate performance, fast charge transfer capability and small equivalent series resistance (ESR).

SPC was chosen for EIS test to analyze electrical resistance property. The testing frequency range is 0.01 Hz-100 kHz with an alternate current amplitude of 5 mV. The value of ESR is obtained from the intercept between the ordinate origin and the starting point of the Nyquist plot at horizontal axis. It can be clearly seen from Fig. 5c that the ESR of SPC is nearly 0.19, which is lower than the majority biomass-based PCs^5,8,42–44^. The low value of ESR can be partly attributed to the developed micropore structure. According to the former studies^45,46^, underdeveloped micropore structure has inhibition effect at the process of electrolyte penetration. What’s more, the two-step acid pickling can remove most of the impurities, which can increase the conductivity to some extent. In high frequency region, the curve reaches a perfect semicircle, which is corresponding to the former test, indicate SPC shows an obvious character of EDLC^47^. The simulated equivalent circuit diagram of the tested supercapacitor is shown in Fig. S2a. L1 represents the resistance of wire in the test circuit. The semicircle in high frequency region can be explained by R1, R2, W and C1. R1 is related to the impedance of the electrolyte, R2 is in connection with current exchange, C1 is concerned with Butler-Volmer equation, and Q can explain the oblique line in the low frequency range. What’s more, the fitted curve almost completely coincides with original curve (Fig. S2b), which means the simulation is consistent with the actual circuit.

Rate performance and cycle life are critical factors for the supercapacitors in application. According to Fig. 5d, all the PC samples show high specific capacitance at the current density of 0.1 A g^−1^. Especially for the OPC, its specific capacitance reaches 568 F g^−1^. When the test current density increased to 5 A g^−1^, all the specific capacitances of the samples are sharply decreased. But the specific capacitance turns to be stable when the current density keeps increasing. That is because at high current density, it is hard for some small micropores to form electrochemical double layers^48–51^. Therefore, with the current density keeps increasing, the unused portion of these pores are increased, and there is a decrease of the capacitance.

It can be seen from Table S2 that at the current density of 10 A g^−1^, the specific capacitance of SPC, GSPC, GPC, OPC are 289 F g^−1^, 320 F g^−1^, 305 F g^−1^, and 323 F g^−1^, respectively. At the current density of 50 A g^−1^, the values become 245 F g^−1^, 279 F g^−1^, 239 F g^−1^, and 294 F g^−1^. The good rate capability can be attributed to reasonable pore structure, and OPC shows obvious advantage at high current density. Considering Table 1, OPC has higher pore volume and higher mesopore volume, which gives it a slightly superior performance over other samples. Compared with other biomass-based PCs shown in Table 2, the PC produced in this study show both advantage in SSA and specific capacitance.

**Table 2:** SSA and specific capacitance of some biomass-based PC for supercapacitors.

Raw material	SSA (m^2^ g^−1^)	Electrolyte	Specific capacitance (F g^−1^@0.5 A g^−1^)	Ref.
Soybean root	2143	6 M KOH	276	^67^
Auricularia	1103	6 M KOH	374	^68^
Tobacco rods	2097	CH_3_(CH_5_)_3_NBF_4_	144	^69^
		6 M KOH	287	
Onion husks	2571	TEABF_4_	188	^70^
Banana fibers	1097	1 M Na_2_SO_4_	74	^40^
Popcorn	1489	6 M KOH	245	^71^
Ant powder	2650	1 M Na_2_SO_4_	273	^72^
		EMIMBF_4_	238	
Garlic seedling	2370	6 M KOH	387	This work
Garlic sprout	2659	6 M KOH	405	
Onion	2342	6 M KOH	395	
Scallion stalk	3154	6 M KOH	343	

Long-term stability is an important factor for energy storage element. After 5000 cycles (Fig. 5e), the capacitance retention are: 92%, 92%, 97%, and 85% for SPC, GSPC, GPC and OPC, indicating the PC samples both have excellent electrochemical stability and superb reversibility. It should be noted that there are obvious increase for specific capacitance near 1100 cycle because the temperature of electrolyte is increasing as the cycle goes on, which will enhance the diffusion of ion and the reduce the ESR^52^. The degradation of the curves can be attributed to the destruction of the carbon structure and the reduction of some oxygen-containing groups on the surface^42^. However, these two effects are very limited. For application, three cell button made from OPC was connected in series and successfully lighten up a 5 mm LED, as shown in Fig. 5f.

Energy density and power density are common factors to evaluate an energy storage device. All the 4 samples have high energy density and power density according to Fig. S3. Figure 5g shows the energy density and power density of OPC and some other common energy storage devices^53^. According to Fig. 5d, OPC has obviously higher energy density than common supercapcitors and higher power density than most of the energy storage devices. The maximum energy density can reach 71 W h kg^−1^ @ 180 W kg^−1^, and the maximum power density can reach 210 kW kg^−1^@33 W h kg^−1^. Compared to other studies^38,39,54–57^, the energy density and power density of the PC samples in this work are beyond compare, which confirms the great promise in practical applications.

### Component analysis

**Figure 6:**
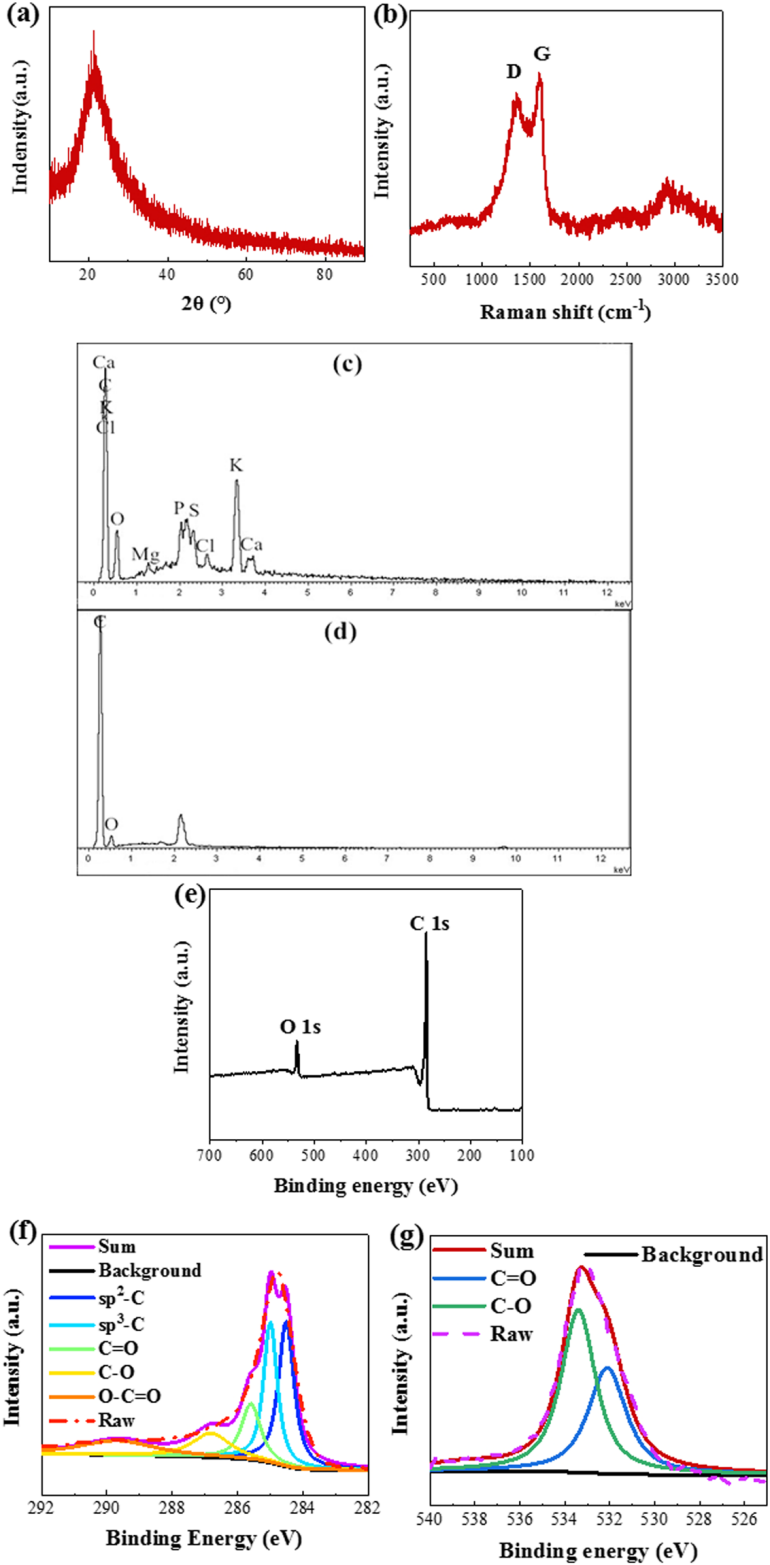
Component analysis of GSPC (**a**) XRD pattern of GSPC (**b**) Raman spectra of GSPC (**c**) EDS spectrum of garlic seeding (**d**) EDS spectrum of GSPC (**e**) XPS spectrum of GSPC.

GSPC was chosen for further component analysis, including the degree of graphitization, element contents, and the types of functional groups. A broad peak near 2θ = 23° can be found in Fig. 6a, which means the dominant component of the material is amorphous carbon. For Fig. 6b, the Raman spectrum show two peaks around 1350 cm^−1^ (D-band) and 1590 cm^−1^ (G-band). The D-band represents the defect of graphitic structure and the disorder of carbon^58,59^, while the G-band means the internal vibration of sp^2^ carbon atoms^60^. The intensity ratio (*I*_G_/*I*_D_) represents the disorder in the carbon matrix^59^, and higher value of (*I*_G_/*I*_D_) means higher degree of graphitization. For GSPC, the value is 1.05, which is higher than commercial PC material (0.52)^8^. Higher degree of graphitization represents higher electrical conductivity and lower electrical resistance. In other words, higher degree of graphitization is benefit for the transfer of the charge, and can directly improve the electrochemical performance^61^. It is speculated that the relatively uniform structure (both the parenchyma and vascular bundle) of the selected precursor can contribute to the formation of the ordered graphite microcrystalline structure.

Figure 6c,d show the elemental composition of precursor and GSPC, more specific data are listed in Tables S3 and S4. According to the results in Fig. 6c, as biomass precursor, the component of GSPC is complicated. Except for the basic element C and O, there are also some essential elements for plants like K, Mg, Ca and P. These elements are impurities for electrode material^62^. It cannot be ignored that the content of K is much higher than other impurity elements, and it can play a very important part during the activation progress. For parenchyma, the K is abundant in the large vacuole. K is also abundant in vascular buddle because it can regulate the osmotic pressure, which is closely related with its transport function. After two-step acid pickling, the majority impurity elements are removed from the final product, and the oxygen is reserved (Fig. 6d).

XPS results (Fig. 6e) also confirmed that the dominant elements of GSPC are carbon and oxygen. The XPS C1s spectrum in Fig. 6f can be de-convoluted by 4 peaks representing sp^2^-bonded carbon (284.6 eV), sp^3^-bonded carbon (285.6 eV), C-O (286.7 eV), and C=O (288.4 eV). The XPS O1s spectrum in Fig. 6g exhibits mainly two peak consisting of C=O (531.6 eV) and C-O (532.4 eV). The oxygen-containing functional groups can improve electrochemical performance by provide additional pseudocapacitance and enhance the wettability of PC material^7,63–65^.

### Preparation principle

**Figure 7:**
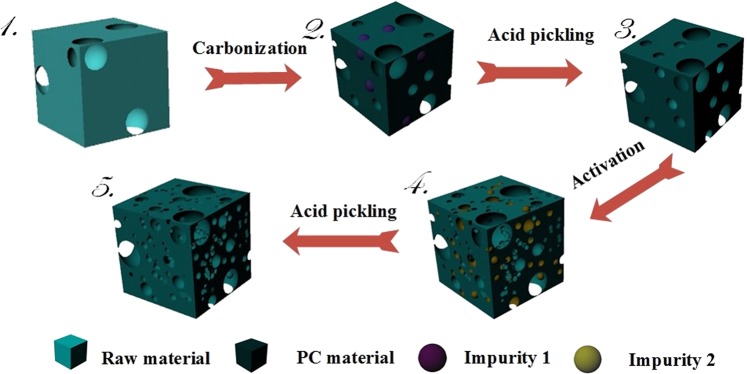
The schematic diagram of PC preparation process.

According to the previous analysis, the schematic of preparation mechanism is shown in Fig. 7. As process 1. shows, there are inherent functional pores (macropores) on the surface of the selected precursors. The initial pore structure, including interspaces and channels, can contribute to sufficient carbonization and activation. During carbonization in process 2., moisture and volatile are released, with the forming of some new pores. Certain essential elements for plant like K, Ca, Mg, and etc. are still in the carbonization product. These elements have bad effect during the energy storage process, and they should be removed after preparation. As shown in process 3., acid pickling and washing after carbonization can remove most of these impurities (impurity 1) and generate new pores in their original position. These pores are mostly macropores and mesopores, and they can promote activation process by provide more contact areas for activator. Most of the pores are formed during activation process, which is usually described by this formula^66^:

5 $$6{\rm{KOH}}+2{\rm{C}}\to 2{{\rm{K}}}_{2}{{\rm{CO}}}_{3}+2{\rm{K}}+3{{\rm{H}}}_{2}$$

This process also followed by the decomposition of K_2_CO_3_ and reaction between the product and byproduct:

6 $${{\rm{K}}}_{2}{{\rm{CO}}}_{3}\to {{\rm{K}}}_{2}{\rm{O}}+{{\rm{CO}}}_{2}$$

7 $${{\rm{K}}}_{2}{\rm{O}}+{\rm{C}}\to 2{\rm{K}}+{\rm{CO}}$$

8 $${{\rm{K}}}_{2}{{\rm{CO}}}_{3}+2{\rm{C}}\to 2{\rm{K}}+3{\rm{CO}}$$

The formation of the pores follows the consumption of C and the formation of the impurities according to process 4. When the temperature exceeds the boiling point of K, the potassium will exist in the form of vapor and interspersed between layers of carbon to create new pores. After the secondary acid pickling and washing, the impurities (impurity 2) generated from activation process are removed, and new pores has formed in relevant position (process 5.). Based on this method, a hierarchical PC with developed pore structure can be produced.

**Figure 8:**
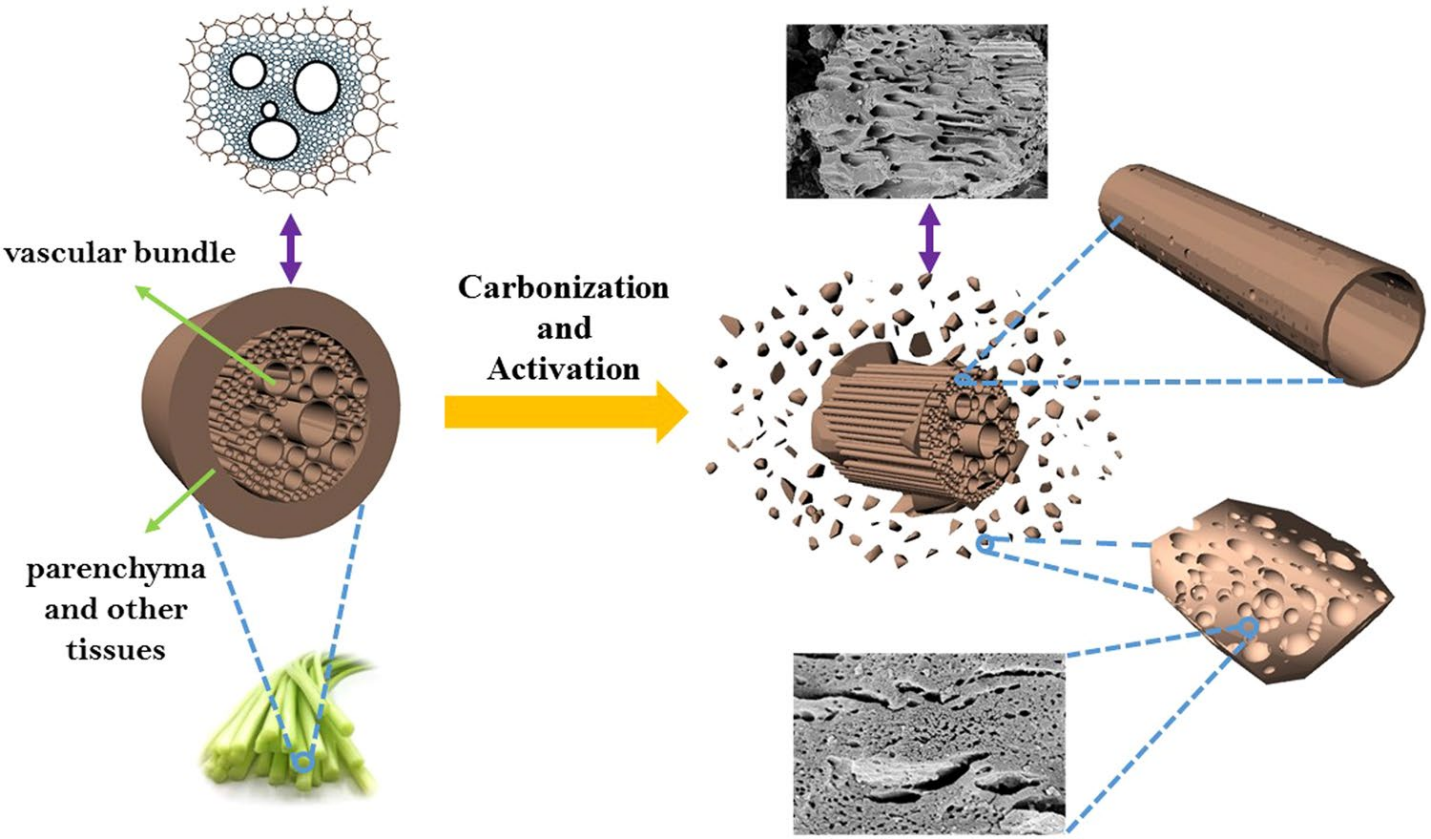
The schematic diagram about evolution of plant tissue during preparation process.

The good structures of PC samples are inherited from the natural structure of plant tissue, and it endows the product with better electrochemical performance. Specific tissue evolution during preparation process is shown in Fig. 8. Combined with SEM results, the vascular bundle is wrapped with cortex before treatment. The carbonization and activation process can easily break the cortex, which is rich in parenchyma, into carbon particles for further reaction. Also, the peeling of the cortex makes vascular structure expose to the activator for further pore-creating process. Vascular bundle is natural transport structure for energy storage, and the activation process creates abundant pores on its surface and its internal channels, which effectively improves its energy storage capability.

## Conclusions

Plants rich in parenchyma tissue and vascular bundle are suitable for preparing hierarchical PC with good electrochemical performance. All the precursors used in this study are abundant in parenchyma tissue and vascular bundle, and the prepared PC samples have similar excellent hierarchical pore structure and electrochemical performance. The evolution of plant tissue during preparation process is discussed, and the mechanism of the preparation method is studied. Two-step acid pickling can effectively remove most of the impurities, and it can both create more pores and lower the ESR of PC. The maximum SSA of these four samples can reach 3154 m^2^ g^−1^. Based on good hierarchical porous structure, the electrochemical performance of the PC materials are excellent. The specific capacitance can reach 568 F g^−1^ at the current density of 0.1 A g^−1^ with obvious EDLC characters. It also has lower ESR, good cycle performance and rate capability, high power density and energy density. This study provides a new perspective for selecting biomass precursor for PC, and the superior electrochemical performance endows the huge potential for large-scale production.

## Supplementary information

Supplementary Information

## Data Availability

The data used to support the findings of this study are included within the article and the supporting information.
