# Supplementary material for: High performance hierarchical porous carbon derived from distinctive plant tissue for supercapacitor
Source: Sci Rep. 2019 Nov 21;9:17270. doi: 10.1038/s41598-019-53869-w (PMC6872525; doi:10.1038/s41598-019-53869-w)
Supplement: Supplementary file 1 — Supplementary Information [file 41598_2019_53869_MOESM1_ESM.pdf]

Supporting Information

# High performance hierarchical porous carbon derived from distinctive plant tissue for supercapacitor

## Authors

Jinxiao Li, Yang Gao, Kuihua Han [z], Jianhui Qi, Ming Li, Zhaocai Teng

( School of Energy and Power Engineering, Shandong University, 250061 Jinan, PR China)

([z]Email：hankh@163.com;

Address: No.17923 Jingshi Road, Lixia District, Jinan, Shandong Province 250061, P.R. China; Tel: +8613589033524, Fax: +86-531-88392414)

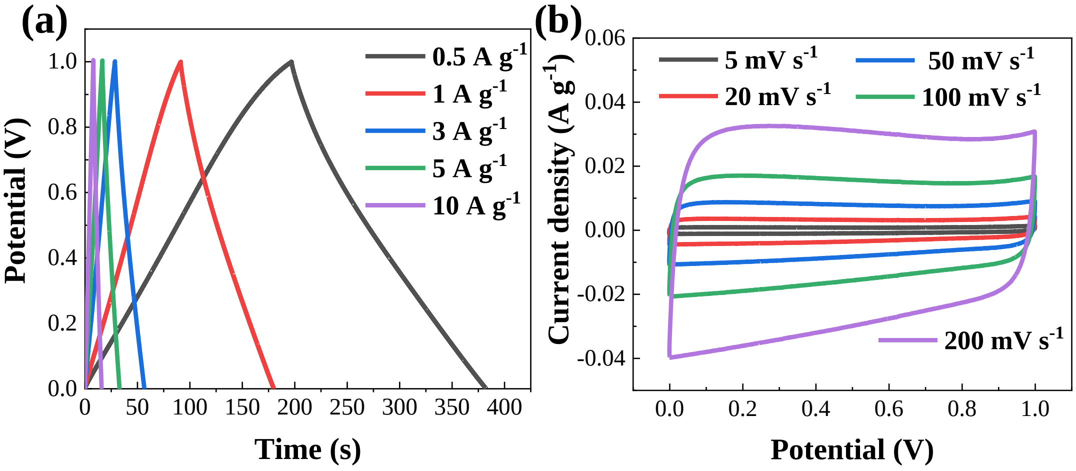


**Figure S1.** GCD and CV curves of OPC (a) GCD curves of OPC at different current density (b) CV curves of OPC at different scan rate

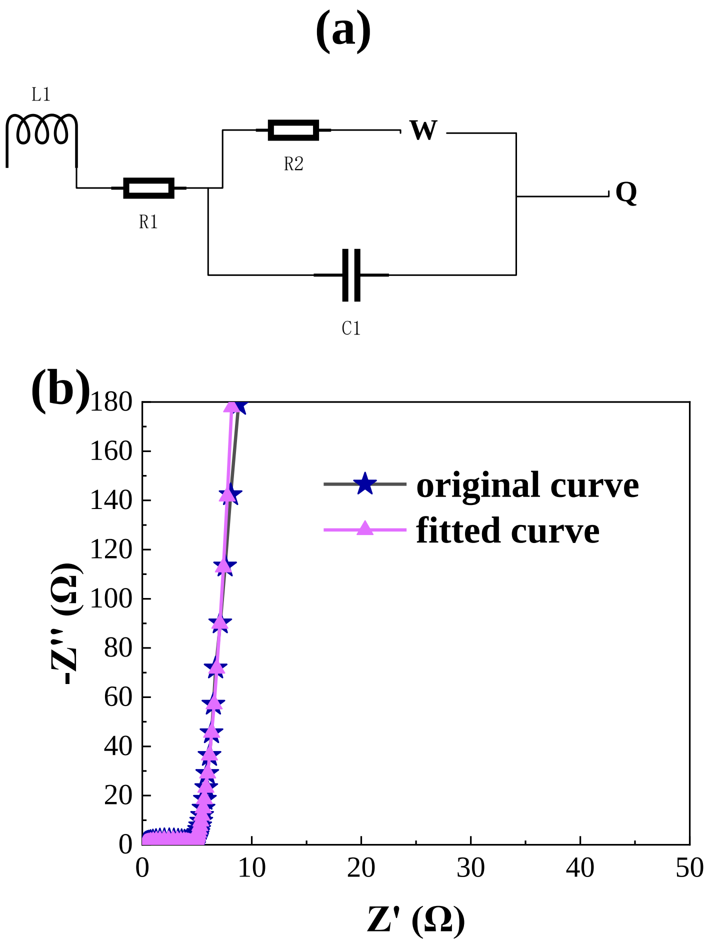


**Figure S2.** Simulated result of equivalent circuit (a) The equivalent circuit diagram of tested supercapacitor made by SPC (b) The comparison diagram between fitting curve and the original curve

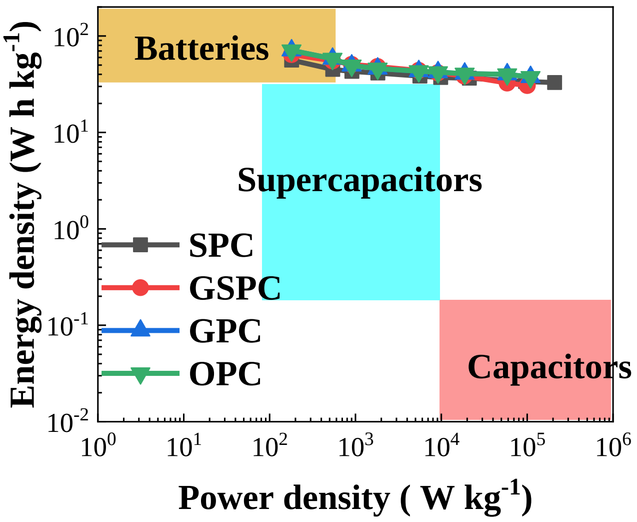


**Figure S3.** Power density and Energy density of 4 PC samples

**Table S1** Characteristics of pores in carbonized garlic sprout before and after acid pickling

| Sample | SSA ($m^2$ $g^{-1}$) | $V_T$ ($cm^3$ $g^{-1}$) | $V_{micro}$ ($cm^3$ $g^{-1}$) | D (nm) | $D_{micro}$ (nm) |
|---|---|---|---|---|---|
| Before | 7.70 | 0.084 | — | 9.91 | — |
| After | 56.26 | 0.116 | 0.022 | 5.04 | 0.40 |

**Table S2** Specific capacitance under different current density

| Sample | Current density (A $g^{-1}$) | | | | |
|---|---|---|---|---|---|
| | 0.1 | 1 | 5 | 10 | 50 |
| GSPC | 513 | 364 | 334 | 321 | 279 |
| GPC | 514 | 385 | 331 | 305 | 239 |
| OPC | 568 | 367 | 335 | 323 | 294 |
| SPC | 450 | 330 | 296 | 289 | 245 |

**Table S3** EDS analysis of garlic seedling

| Element | Wt% | At% |
| --- | --- | --- |
| C K | 10.57 | 18.44 |
| O K | 41.21 | 53.99 |
| Mg K | 1.55 | 1.34 |
| P K | 3.88 | 2.63 |
| S K | 4.59 | 3.00 |
| Cl K | 3.45 | 2.04 |
| K K | 29.60 | 15.87 |
| Ca K | 5.14 | 2.69 |
| Matrix | Correction | ZAF |

**Table S4** EDS analysis of GSPC

| Element | Wt% | At% |
| --- | --- | --- |
| C K | 52.3 | 59.36 |
| O K | 47.7 | 40.64 |
| Matrix | Correction | ZAF |
